# Unveiling the driving role of pH on community stability and function during lignocellulose degradation in paddy soil

**DOI:** 10.3389/fmicb.2024.1338842

**Published:** 2024-02-26

**Authors:** Yi Wang, Yonglun Chen, Xiuqing Gao, Qiong Wang, Mingyu Cui, Dongdong Zhang, Peng Guo

**Affiliations:** ^1^Institute of Agricultural Products Processing and Nuclear Agriculture Technology Research, Hubei Academy of Agricultural Sciences, Wuhan, China; ^2^Sichuan Jiahuai Biotechnology Co., Ltd., Leshan, China; ^3^College of Biological and Pharmaceutical Sciences, Three Gorges University, Yichang, China; ^4^Institute of Marine Biology, Ocean College, Zhejiang University, Zhoushan, Zhejiang, China; ^5^Hubei Hongshan Laboratory, Wuhan, China

**Keywords:** paddy soil, straw incorporation, bioaugmentation, biostimulation, microbial community, pH influence

## Abstract

**Introduction:**

Crop straw, a major by-product of agricultural production, is pivotal in maintaining soil health and preserving the ecological environment. While straw incorporation is widely recognized as a sustainable practice, the incomplete decomposition of crop residues poses challenges to plant growth, increasing the risk of pests and diseases. This necessitates a comprehensive investigation.

**Methods:**

The current study employs a 28-day pot experiment to simulate the degradation of rice straw in paddy soils. The impacts of bioaugmentation and biostimulation on lignocellulose degradation are systematically evaluated.

**Results:**

Results indicate a high lignocellulose degradation ability in paddy soil, with over 80% straw weight loss within 28 days. Bioaugmentation with a lignocellulolytic microbial consortium enhances straw degradation during the initial stage (0–14 days). In contrast, biostimulation with readily available nutrients leads to soil acidification, hindering straw degradation and reducing microbial diversity. Furthermore, pH emerges as a critical factor influencing microbial community stability and function during lignocellulose degradation. Microbial co-occurrence network analysis reveals that microorganisms occupy ecological niches associated with different cellulose components. Notably, Module M2, comprising Proteobacteria, Firmicutes, Gemmatimonadota, Actinobacteriota, Bacteroidota, Myxococcota, Halobacterota, and Acidobacteriota, positively correlates with pH and weight loss.

**Discussion:**

This study significantly advances our understanding of microbial mechanisms in soil decomposition, emphasizing the pivotal role of pH in community stability and function in paddy soil. These findings can inform future strategies for managing rice straw while safeguarding soil ecosystem health.

## 1 Introduction

Crop straw is a major by-product of agricultural production, and its management is crucial for soil health and the ecological environment (Lu et al., 2020). In conventional farming, straw is often burned in the field to facilitate the planting of the next crop. While ash from straw increases the soil pH and mineral ion content (Walter et al., 1996), burning straw releases large amounts of anthropogenic greenhouse gases (GHGs) into the air (Ren et al., 2019). Conversely, straw incorporation is a sustainable alternative that not only avoids air pollution caused by burning but also prevents soil degradation and increases soil organic carbon (Wang et al., 2018).

In order to facilitate the sustainable advancement of agriculture, China is actively encouraging the practice of returning crop straw to the fields. Incorporating straw into cultivated soils can promote the development of a favorable soil environment and alleviate soil degradation caused by intensive and continuous conventional tillage practices (Yin et al., 2018). Straw incorporation also helps regulate nutrient pools, including nitrogen, phosphorus, and sulfur pools, ultimately improving soil fertility and stimulating the growth of beneficial microorganisms (Wang et al., 2018). In tropical and temperate regions, straw incorporation increases soil microbial biomass significantly (Bu et al., 2020; Cong et al., 2020); however, these benefits are contingent on the proper decomposition of crop residues. Meanwhile, improper management or careless disposal of straw in the field remains a significant issue. Undecomposed straw can impede plant growth and increase the risk of pests and diseases affecting crops (Goswami et al., 2020; Tang et al., 2020; Wang X. et al., 2023).

Straw incorporation significantly affects the structure of soil microbial communities, leading to increased network complexity and ecological stochasticity (Xu et al., 2023). Moreover, microorganisms play critical roles in soil functions including soil respiration and the decomposition of organic matter (Li et al., 2021). Although the effects of straw incorporation on soil microorganisms have been investigated (Bu et al., 2020; Cong et al., 2020), straw decomposition in the soil has received limited attention. Complete straw degradation can take several years, particularly under poor soil conditions (Wang et al., 2022) as plants have developed complex structural and chemical mechanisms to resist microbial degradation of their structural sugars (Himmel et al., 2007). To accelerate the decomposition of straw, agricultural machinery operations, such as crushing and plowing, are employed, along with the application of microbial agents and organic fertilizers (Goswami et al., 2020). Bioaugmentation using microbial agents enhances lignocellulose degradation by introducing lignocellulolytic microbes from external sources (Wang Y. et al., 2023). Conversely, organic fertilizers act as biostimulants by promoting the growth and activity of native microorganisms by providing essential nutrients (Tang et al., 2020).

Although the use of microbial agents and organic fertilizers is widespread in straw incorporation, their interactions with indigenous microorganisms remain poorly understood. One crucial concern is the eutrophication of the environment created by the return of straw and organic fertilizers to the soil. Freshly incorporated straw and inadequately decomposed organic fertilizers introduce substantial amounts of available carbon and nitrogen into the soil. This results in the generation of ecological niches for the growth of soil microorganisms (Bastida et al., 2021; Liu B. et al., 2021). However, an unbalanced supply of nutrients can affect the equilibrium of the microbial community structure. According to the “Black Queen Hypothesis” (Morris et al., 2012; Oliveira et al., 2014), an excess of public goods can trigger retrogressive evolution within microbial communities. In nutrient-rich conditions, opportunistic individuals gain a competitive advantage due to their reduced metabolic burden of producing public goods. Consequently, nutrient-rich conditions decrease microbial diversity and affect the multifunctionality of the microbiome (Finn et al., 2022). It is, therefore, essential to recognize the potential adverse effects of organic fertilizers on straw degradation.

This study aims to enhance the current understanding regarding straw decomposition in soil, elucidate the underlying microbiological mechanisms, and provide valuable guidance for effective straw management practices. To this end, pot experiments were conducted to simulate straw decomposition in paddy soil. The effects of bioaugmentation and biostimulation were assessed following the introduction of a lignocellulolytic microbial consortium and available nutrients. As such the significance of this study is threefold as it (1) assesses the impact of incorporating cellulose-degrading bacteria from external sources and providing readily available nutrients on straw decomposition; (2) explores the succession of the soil microbial community during this process; and (3) investigates the relationship between the soil microbial community structure and soil function while identifying the underlying driving factors.

## 2 Materials and methods

### 2.1 Experimental design

Soil samples were collected from depths of 0 to 20 cm from a dried paddy field in July 2020, at Wuhan (30°29′4^′′^ N, 114°18′53^′′^ E), Hubei Province, China. The soil type was fluvo-aquic with a pH of 7.50, containing 1.97% organic matter, 0.21% total nitrogen, and 0.03% nitrate-nitrogen. The soil samples were homogenized and sifted through a 2-mm mesh to remove rocks and plant tissues from the field. During this season, rice straw was collected after harvesting and cut into 5 cm pieces after air-drying. To facilitate the observation of lignocellulose degradation, rice straw was alkali-treated before use (Guo et al., 2011).

**Figure 1:**
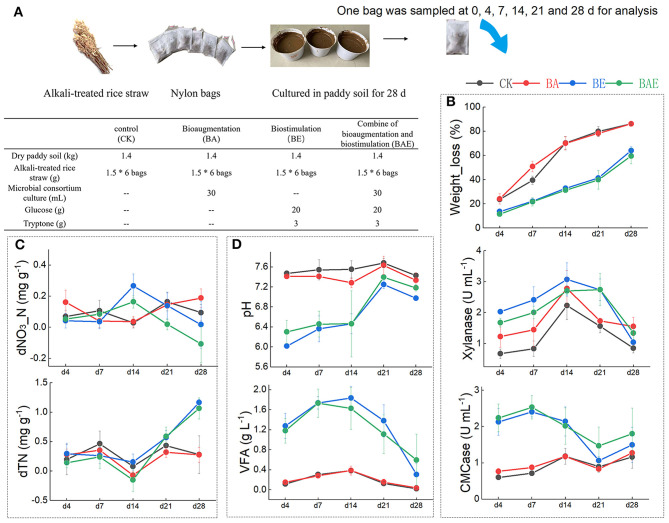
Effect of various treatments on rice straw degradation in paddy soil. **(A)** Experimental design. **(B)** Weight loss of the rice straw and the relative enzymic activities; **(C)** soil nitrogen; **(D)** soil acidity. The data represent the mean and standard deviation from triplicate independent experiments.

Four groups of treatments were designed (Figure 1A); each treatment was performed in triplicate. The experiment was performed in pots containing 1.4 kg of air-dried paddy soil. The bioaugmentation (BA) group was treated with 30 mL of lignocellulolytic consortium XDC-2 culture, which is effective for the degradation of natural rice straw over a wide range of temperatures, and mainly composed of mesophilic bacteria related to the genera Clostridium, Bacteroides, Alcaligenes, Pseudomonas, etc. (Guo et al., 2010; Hui et al., 2013). The BE group was biostimulated with readily available nutrients: 5 g of glucose and 2 g of tryptone. The BAE group underwent bioaugmentation with 30 mL of XDC-2 culture and biostimulation with readily available nutrients. The CK group served as the control with no additional treatments. All treated samples were then added to 500 mL of sterile water and sufficiently mixed. To investigate the *in situ* decomposition of straw in soil while minimizing the impact of sampling on the system, we employed nylon bags in our experiment. Specifically, six independent nylon bags containing 1.5 g of alkali-treated rice straw were added to 10 g of the mixed soil sample and potted at 5–15 cm depth. The total inoculation time was 28 d, and the nylon bags were collected at 0, 4, 7, 14, 21, and 28 d for analysis.

### 2.2 Chemical analyses

#### 2.2.1 Degradation of rice straw

The straw was measured by the gravimetric method after being washed and oven-dried at 80°C to a constant weight. Lignin (LG), soluble substances (SS), cellulose (Cel), and hemicellulose (HC) in the residual rice straw were analyzed using an ANKOM220 fiber analyzer (ANKOM Technologies, USA) as previously described (Guo et al., 2011).

#### 2.2.2 Soil acidity

The soil pH was measured using a pH meter (Mettler PE28, Switzerland) in 1:2 (w/v) soil:water suspensions. The concentration of volatile fatty acids (VFA) was determined using a colorimetric method based on a ferric hydroxamate reaction (García et al., 2020). Briefly, 0.4 mL of prepared sample was mixed well with 3 mL of ethylene glycol containing 10% sulfuric acid. After boiling at 100°C for 10 min, the mix was cooled to room temperature and added in order with 0.4 mL of 10% (w/v) hydroxylammonium chloride, 0.4 mL of 4.5 M sodium hydroxide, and 2 mL of 1.2% ferric chloride containing 10% sulfuric acid. After mixing well, the sample was incubated for 5 min, and the optical density was measured using a BioMate 160 UV-VIS spectrophotometer (ThermoFisher, USA) at a wavelength of 495 nm.

#### 2.2.3 Soil nitrogen

Total nitrogen (TN) was determined using the Kjeldahl method (Nelson and Sommers, 1980). Nitrate–nitrogen contents (NO_3__N) were measured using an AutoAnalyzer3 continuous flow analyzer (Seal Analytical, Germany). To account for the additional nitrogen introduced into the soil by adding readily available nutrients, we calculated dTN (change in total nitrogen) and dNO_3__N (change in nitrate–nitrogen) relative to their initial contents.

### 2.3 Extracellular enzyme assay

Xylanase (EC 3.2.1.8) and cellulase (EC 3.2.1.4) activities (CMCase) were assayed at 40 °C to evaluate the capability of the soil to degrade lignocellulosic materials (Hui et al., 2013); beechwood xylan and sodium carboxymethylcellulose were used as substrates, respectively. The released soluble reducing sugars, xylose and glucose, were measured at 520 nm using the 3,5-dinitrosalicylic acid (DNS) method (Hui et al., 2013).

### 2.4 High-throughput sequencing

For each sample, total DNA from fresh soil (0.5 g) was extracted using an E.Z.N.A. Soil DNA kit (Omega, USA). The quantity and purity of DNA were determined using NanoDrop2000 microspectrophotometry (Thermo Fisher Scientific, USA). The V3–V4 region of bacterial the 16S rRNA was amplified using the 341F (5′-CCTAYGGGRBGCASCAG-3′) and 806R (5′-GGACTACNNGGGTATCTAAT-3′) primers (Qiu et al., 2021). The samples were sequenced on an Illumina MiSeq PE300 platform (Majorbio, China) in a paired-end manner.

### 2.5 Bioinformatics and statistical analysis

For each analysis, the average of triplicate results (mean ± SD) was used, and results with *P* < 0.05 were considered significant unless specified otherwise. Bioinformatic analysis was performed using the Majorbio Cloud Platform online tool (Ren et al., 2022). Raw sequencing reads were deposited in the NCBI Sequence Read Archive (SRA) database under accession number PRJNA1010947. To correct for uneven sequencing, rare OTUs with < 20 reads were removed, and the abundance was normalized according to the lowest sequence number.

Further correlation analyses were performed using the “microeco” package in R (version 4.3.0). Correlations among the environment variables were assessed and visualized using functions under “trans_env class” based on Spearman's correlation (Liu C. et al., 2021). Construction of the co-occurrence networks and calculation of the topological parameters were performed using functions under “trans_network class” based on Spearman's correlation matrices among microbes. Network visualization was performed using Gephi v 0.9.3 (Bastian et al., 2009).

Structural equation modeling (SEM) was performed using the lavaan R package (Rosseel, 2012) to explore the relationships between environmental variables and soil bacterial communities. A non-significant chi-square test was used to evaluate the model fit. We first considered a full model comprising all reasonable pathways and sequentially eliminated non-significant pathways until the final model achieved a good fit. The evaluation criteria included *P* > 0.05, a chi-square ratio (χ^2^/df) < 3, goodness of fit (GFI) > 0.90, comparative fit (CFI) > 0.90, and root mean square error of approximation (RMSEA) < 0.08 (Li et al., 2021).

## 3 Results

### 3.1 Effect of various treatments on rice straw degradation in paddy soil

The rice straw weight loss revealed that bioaugmentation with exotic lignocellulolytic microbes accelerated the degradation of rice straw during the initial 14 days (Figure 1B). Subsequently, straw degradation in the control group gradually reached that in the BA group, with both groups achieving 80% weight loss. In contrast, the BE and BAE groups exhibited consistently lower rice straw degradation ratios than the control groups throughout the process. Changes in the LG, SS, Cel, and HC components were consistent with rice straw weight loss (Supplementary Figure 1). Corresponding to the weight loss of rice straw, the BA group demonstrated higher xylanase and CMCase activities than the CK group. Unexpectedly, the xylanase and CMCase activities in the BE and BAE groups were higher than those in the CK and BA groups (Figure 1B).

The BA group exhibited minimal differences from the CK group in total nitrogen and nitrate–nitrogen content. In contrast, adding readily available nutrients increased total nitrogen accumulation and decreased nitrate–nitrogen content (Figure 1C). Notable acidification was observed following the addition of readily available nutrients. Moreover, a substantial amount of VFA was generated in the BE and BAE groups, resulting in a marked reduction in pH to 6.0 and 6.4, respectively (Figure 1D). After 14 days, acidification gradually eased owing to the consumption of VFA.

### 3.2 Shifts in the taxonomy of paddy soil bacteria

**Figure 2:**
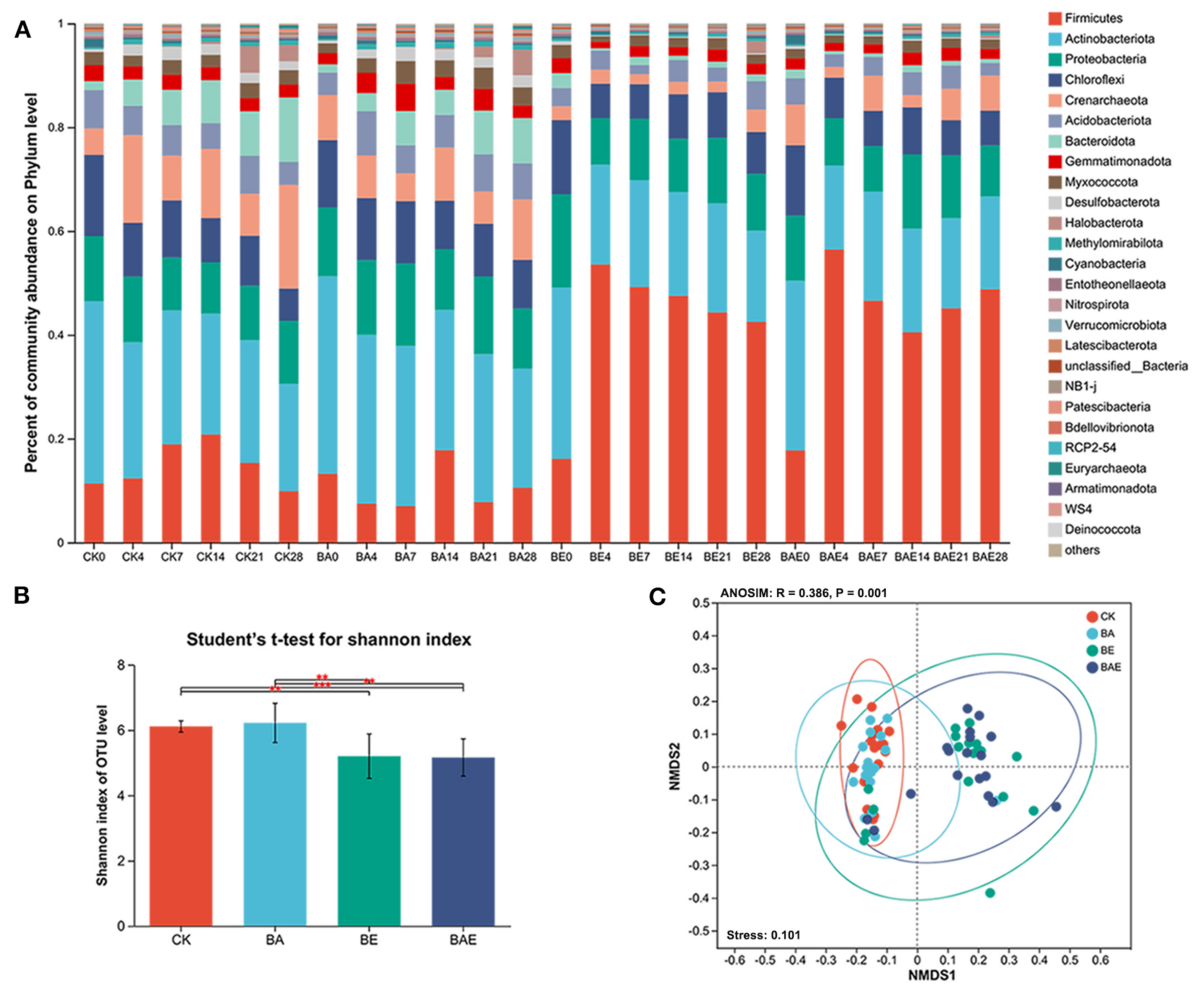
Shift in paddy soil bacteria under various treatments. **(A)** Taxonomy of paddy soil bacteria. **(B)** Alpha diversity differences across various treatments. Student's *t*-test: **0.001 < *P* ≤ 0.01; ***P ≤ 0.001; **(C)** Nonmetric multidimensional scaling (NMDS) based on Bray–Curtis distance, along with Adonis analysis of the complete data set.

In total, 6,489,718 high-quality sequences were obtained after quality filtering and assignment. After removing rare OTUs, 2,862 remained, representing 42 phyla, 690 genera, and 1,254 species. Taxonomic analysis revealed that the five most abundant phyla accounted for > 80% of paddy bacteria: Firmicutes (27.11%), Actinobacteria (24.21%), Proteobacteria (12.43%), Chloroflexi (9.32%), and Crenarchaeota (7.59%; Figure 2A).

Adding readily available nutrients increased the abundance of Firmicutes while decreasing that of Actinobacteria, Crenarchaeota, and Bacteroidetes. The Student's *t*-test for the Shannon index indicated that the addition of readily available nutrients significantly reduced the alpha diversity of the soil bacteria. However, there was little change in alpha diversity after bioaugmentation (Figure 2B).

Nonmetric multidimensional scaling (NMDS) based on Bray–Curtis distance, along with ANOSIM/Adonis analysis of the complete dataset, revealed variation in the bacterial community across different treatments (R = 0.386; Stress = 0.101; Figure 2C). Moreover, ANOSIM/Adonis analysis showed that adding readily available nutrients led to the separation of bacterial community clusters, with R = 0.551 between CK and BE, R = 0.668 between CK and BAE, and R = 0.593 between BA and BAE (Table 1). Distinct clusters were not formed after bioaugmentation, with R = 0.090 between CK and BA and R = −0.0056 between BE and BAE.

**Table 1:** ANOSIM/Adonis analysis.

**Group**	**ANOSIM** ^**a**^			**Adonis** ^**b**^		
	**R** ^c^	***P***	**Permutation_num**	**F. model**	**R** ^2d^	**Pr (**>**F)**^e^
CK vs. BA vs. BE vs. BAE	0.386	0.001	999	10.030	0.307	0.001
CK vs. BA	0.090	0.025	999	2.194	0.061	0.044
CK vs. BE	0.551	0.001	999	13.421	0.283	0.001
CK vs. BAE	0.688	0.001	999	17.674	0.342	0.001
BA vs. BE	0.436	0.001	999	11.212	0.248	0.001
BA vs. BAE	0.593	0.001	999	15.712	0.316	0.001
BE vs. BAE	−0.0056	0.475	999	0.950	0.027	0.395

^a^Analysis of similarities (ANOSIM) was performed based on Bray-Curtis distances.

^b^Adonis is a nonparametric statistical method.

^c^An R value closer to 1 indicates a greater difference between groups.

^d^The R^2^ value shows the percentage of variation explained by the supplied mapping file category.

^e^Pr (>F) is the p-value representing statistical significance.

Permuation_num, number of permutations.

**Figure 3:**
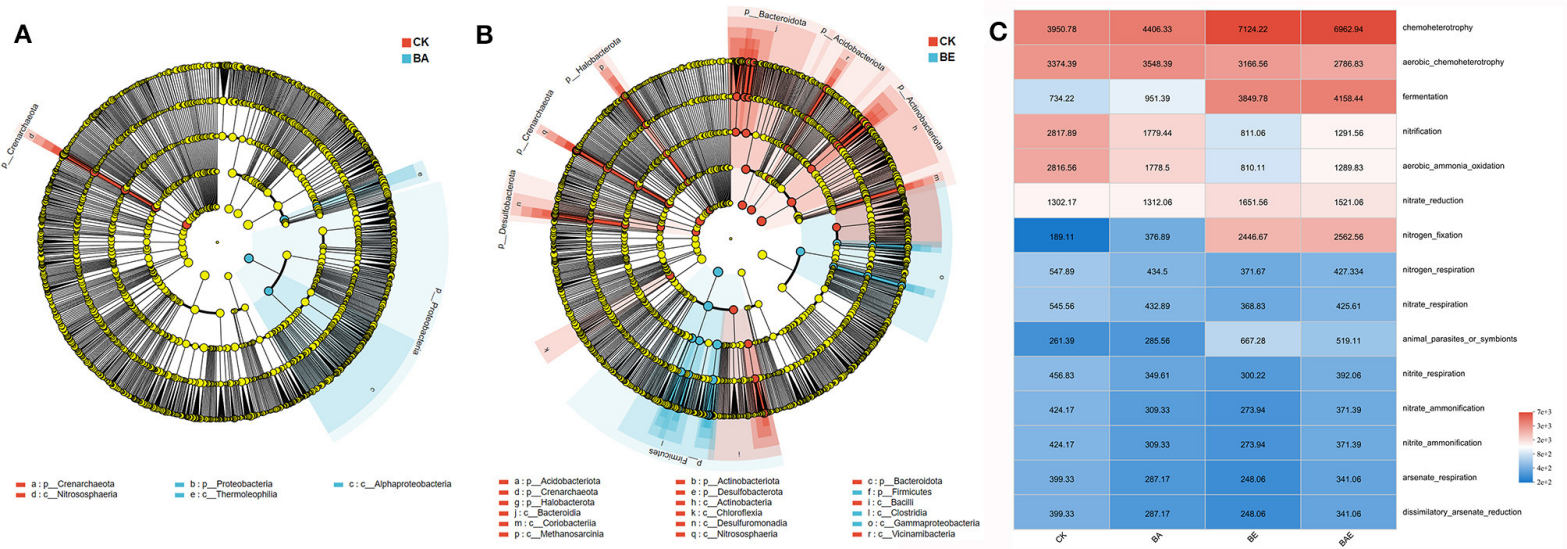
Characteristics of different microbes based on LEfSe analysis **(A, B)** and bacterial function prediction analyses using FAPROTAX **(C)**.

Differential microorganisms were assessed using LDA effect size (LEfSe) analysis. Corresponding to the ANOSIM/Adonis analysis, only a few distinct microbial taxa were observed in the BA and CK groups. The phyla Crenarchaeota and class Nitrososphaeria were enriched in the CK group, whereas the phyla Proteobacteria and class Thermoleophilia were enriched in the BA group (Figure 3A). In contrast, a significant number of different microbial taxa were found in the BE and CK groups (Figure 3B). The addition of readily available nutrients resulted in the enrichment of classes Clostridia and Gammaproteobacteria, both belong to the phylum Firmicutes. However, a considerable number of microbial taxa, including the phyla Acidobacteria, Actinobacteria, Bacteroidetes, Crenarchaeota, Desulfobacterota, Halobacterota, and Actinobacteria were enriched in the control group.

Bacterial function prediction analyses using the Functional Annotation of Prokaryotic Taxa (FAPROTAX) (Louca et al., 2016) revealed that the six most abundant functional groups accounted for > 70% of the total groups (Figure 3C): chemoheterotrophy, aerobic chemoheterotrophy, fermentation, nitrification, aerobic ammonia oxidation, nitrate reduction, and nitrogen fixation. Minimal differences were observed in the functional profiles of the bioaugmentation and control groups. However, the addition of readily available nutrients enhances chemoheterotrophy, fermentation, nitrate reduction, and nitrogen fixation, while reducing aerobic chemoheterotrophy, nitrification, and aerobic ammoniaoxidation (Figure 3C).

### 3.3 Correlation analysis among soil biochemical factors and straw degradation

The degradation of rice straw is expected to be facilitated by extracellular enzymes produced by soil microbes. However, unexpectantly, analysis across all samples revealed a negative correlation between weight loss and CMCase (ρ = −0.452; *P* < 0.001) and a non-significant negative correlation between weight loss and xylanase (ρ = −0.169; *P* > 0.05). This abnormal phenomenon is intriguing and unexpected. Given the pronounced acidification observed after adding readily available nutrients, we examined the correlation between pH and rice straw degradation. A strong positive correlation was detected between pH and weight loss (ρ = 0.633; *P* < 0.001), corresponding to a strong negative correlation between weight loss and volatile fatty acids (VFA) (ρ = −0.640; *P* < 0.001).

**Figure 4:**
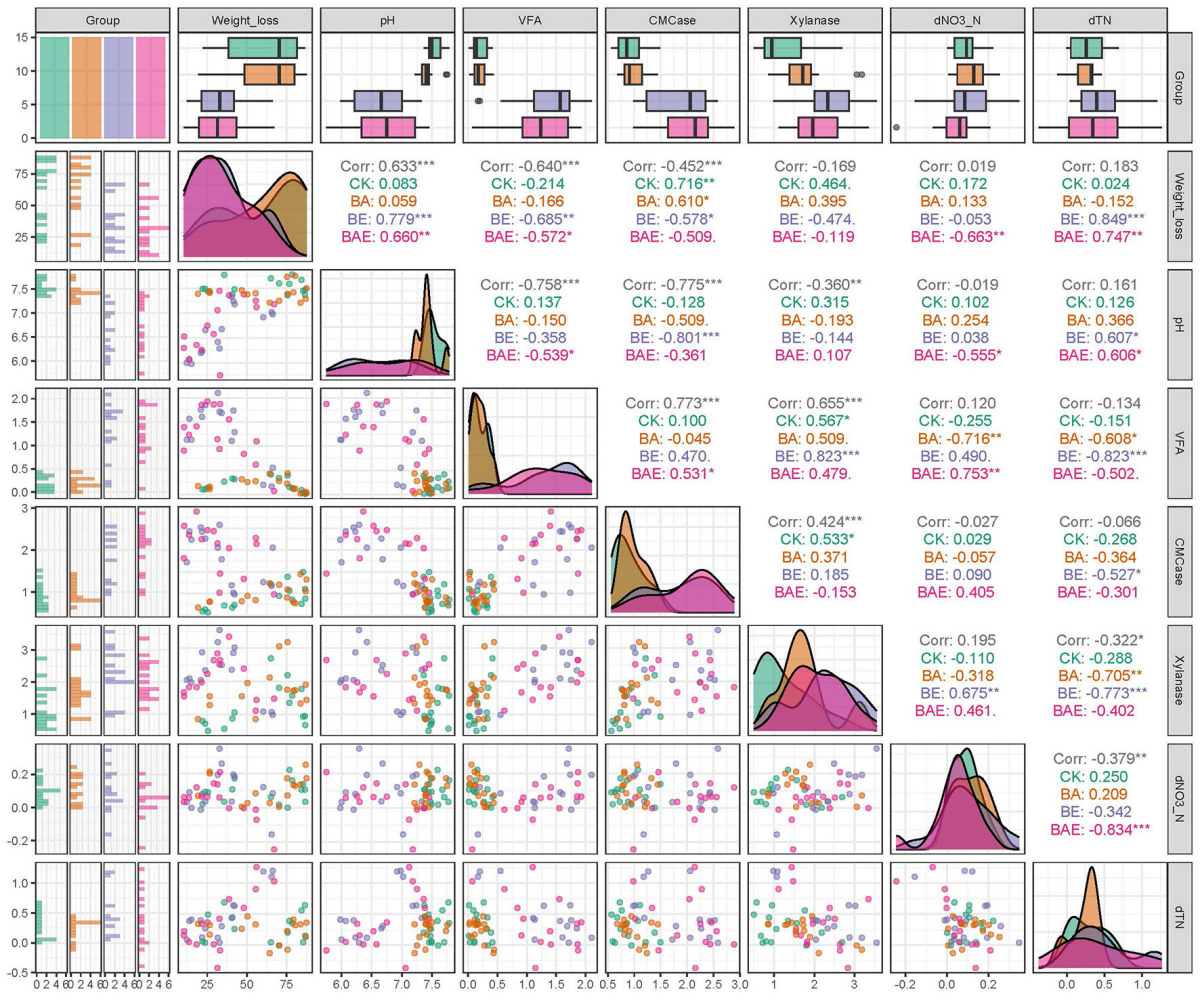
Correlation among soil physicochemical and biochemical variables. Differences were assessed with Student's *t*-test: *0.01 < *P* ≤ 0.05; **0.001 < *P* ≤ 0.01; ***P ≤ 0.001.

When correlation analysis was performed individually for each treatment, CMCase exhibited a positive correlation with weight loss in the CK (ρ = 0.716; *P* < 0.001) and BA (ρ = 0.610; *P* < 0.001) groups. In contrast, CMCase negatively correlated with weight loss in the BE (ρ = −0.57; *P* < 0.05) and BAE (ρ = −0.509; *P* > 0.05) groups. Similarly, xylanase did not significantly correlate with weight loss in the BE (ρ = −0.119; *P* > 0.05) or BAE (ρ = −0.474; *P* > 0.05; Figure 4) groups.

These findings suggest a positive correlation between straw degradation and lignocellulolytic enzyme activity under neutral pH conditions. However, in the presence of acidification, this correlation becomes negative or non-significant. These phenomena highlight the influence of pH on rice straw degradation.

### 3.4 Co-occurrence network between soil microbiota

**Figure 5:**
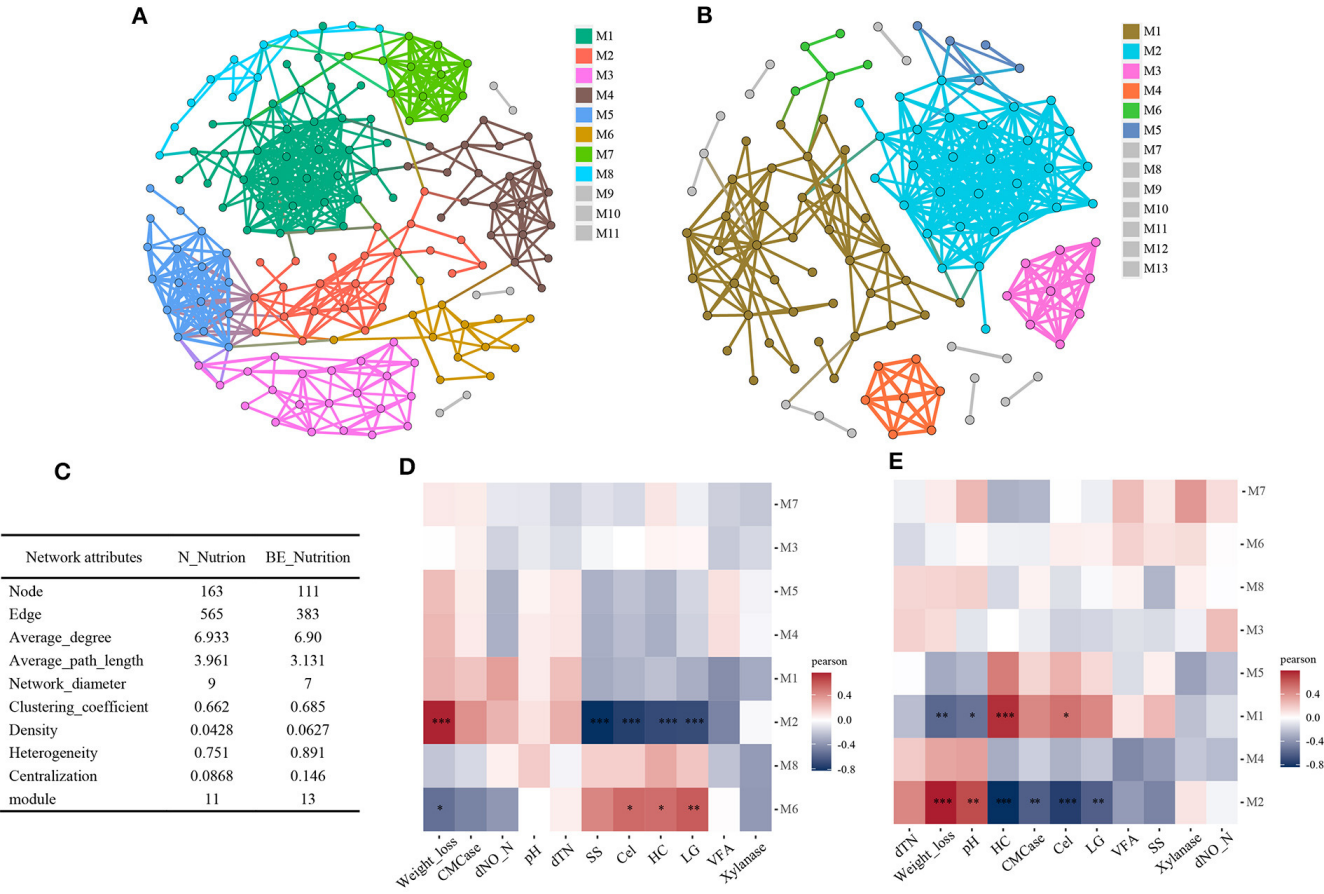
Soil microbiota co-occurrence network analyses according to nutrition addition. High-confidence interactions with Spearman correlation ρ ≥ 0.7 and *P* ≤ 0.01 were retained and displayed as nodes (OTUs) and edges (significant interactions among genera nodes). The same node colors represent genera belonging to the same module. **(A)** Co-occurrence network of natural nutrition group (N_Nutrition), containing the treatment groups CK and BA. **(B)** Co-occurrence network of nutrition addition group (BE_Nutrition), containing BE and BAE treatments. **(C)** Comparison of the modularity and topological features of the co-occurrence network between N_Nutrition and BE_Nutrition. **(D, E)** Correlations between modules and environment variables in the co-occurrence network of the N_Nutrition group **(D)** and BE_Nutrition group **(E)**. *0.01 < *P* ≤ 0.05; **0.001 < *P* ≤ 0.01; ***P ≤ 0.001.

To analyze the effect of microbial interactions on lignocellulose degradation in paddy soil, we conducted a microbial co-occurrence network analysis. Firstly, to distinguish the impact of biostimulation, the data for groups CK and BA were combined to construct a natural nutrition network (Figure 5A), and data for BE and BAE were combined to construct a eutrophic network (Figure 5B). The network demonstrated high-confidence interactions (ρ ≥ 0.7; *P* ≤ 0.01). The modularity and topological features of the co-occurrence network are presented in Figure 5C.

The addition of readily available nutrients significantly affected the structure of the soil microbial network, decreasing the number of nodes (from 163 to 111) and edges (from 565 to 383), indicating a decrease in the number of microbial species and their interactions. The average degree remained relatively constant, whereas the average path length and network diameter decreased, suggesting the formation of a more compact network with nutrient addition. Moreover, the number of modules and clustering coefficient increased, indicating a more modular network structure. The density also increased, indicating more frequent connections between the nodes. Heterogeneity and centralization increased, suggesting that a few highly connected nodes dominated a more centralized network. Overall, these results indicated that the addition of nutrients led to a more compact, modular, and centralized soil microbial network structure.

Associations between eigengenes and environmental factors were calculated and visualized using a correlation heatmap. In the natural nutrition network, module M2 showed a significant positive correlation with weight loss, whereas module M6 was positively correlated with cellulose, hemicellulose, and lignin content (Figure 5D). In the eutrophic network, module M2 exhibited a positive correlation with weight loss and pH and a negative correlation with CMCase, cellulose, hemicellulose, and lignin content. In contrast, module M1 demonstrated a negative correlation with weight loss and pH and a positive correlation with cellulose and hemicellulose content (Figure 5E). Overall, most microbial modules in the natural nutrition and eutrophic networks showed limited correlations with soil biochemical factors. Indeed, this weak differentiation within the groups can be attributed to the grouping based on high- and low-nutrient conditions, which effectively excluded pH as a major factor. Hence, the effect of pH on rice straw degradation may have been masked by nutrient-based grouping, leading to less pronounced differences within the groups.

**Figure 6:**
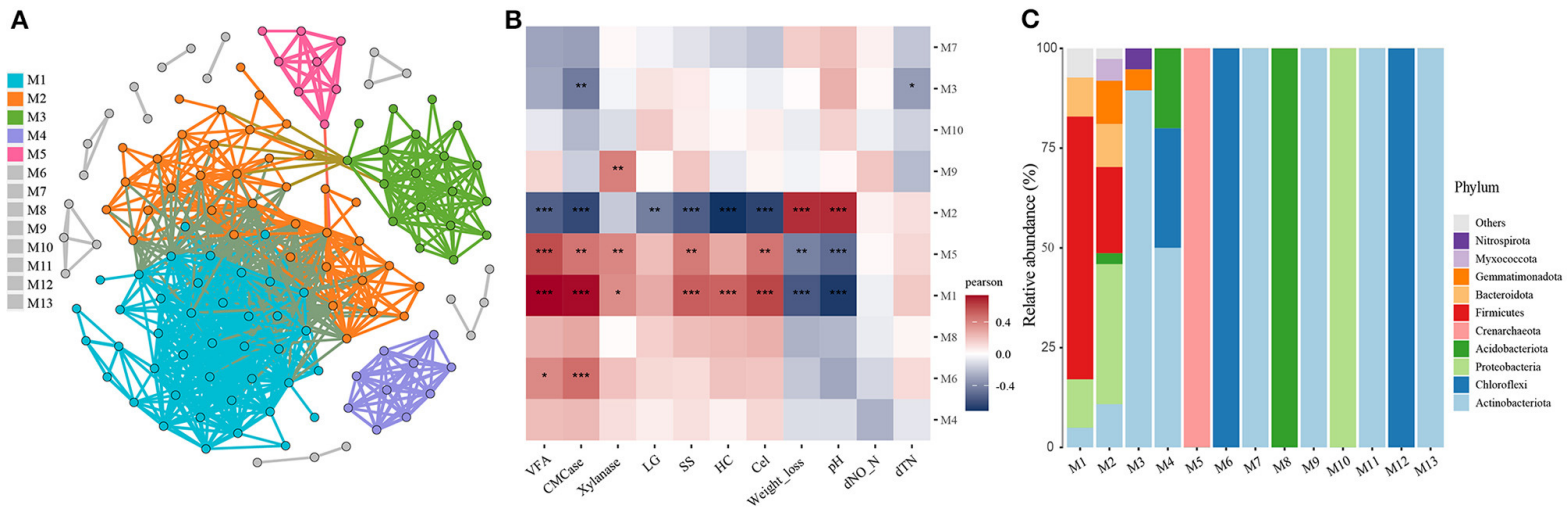
Soil microbiota co-occurrence network analyses across all soil samples. **(A)** Co-occurrence network demonstrated high-confidence interactions with Spearman's correlation coefficient (ρ ≥ 0.7 and *P* ≤ 0.01). All networks are displayed as nodes (OTUs) and edges (significant interactions among genera nodes). The same node colors represent genera belonging to the same module. **(B)** Correlation between modules and environment variables. **(C)** Microbial composition of the modules. *0.01 < *P* ≤ 0.05; **0.001 < *P* ≤ 0.01; ***P ≤ 0.001.

To further investigate the effect of compartment niche selection on soil microbiota and its correlation with biochemical factors, co-occurrence networks were generated across all soil samples (Figure 6A). The resulting network comprised 138 nodes with 865 edges, an average degree of 12.536, and average clustering coefficient of 0.635. The network diameter was 6, and the average path length was 2.243. Thirteen modules were identified, with a modularity index of 0.487. The associations between the modules and soil environmental factors are shown in Figure 6B, along with the microbial composition of the modules displayed in Figure 6C. For instance, modules M1 and M5 exhibited positive correlations with VFA, CMCase, xylanase, cellulose, and soluble substance content and negative correlations with soil weight loss and pH. Module M6 showed positive correlations with VFA and CMCase. Module M9 positively correlated with xylanase activity. Module M3 exhibited a negative correlation with CMCase and dTN. Module M2 exhibited positive correlations with weight loss and pH, and negative correlations with VFA, CMCase, cellulose, hemicellulose, lignin, and soluble substance content. Notably, Module M2 comprised 35.14% Proteobacteria, 21.62% Firmicutes, 10.81% Gemmatimonadota, 10.81% Actinobacteriota, 10.81% Bacteroidota, 5.41% Myxococcota, 2.70% Halobacterota and 2.70% Acidobacteriota.

### 3.5 Environmental drivers of community composition and soil function

**Figure 7:**
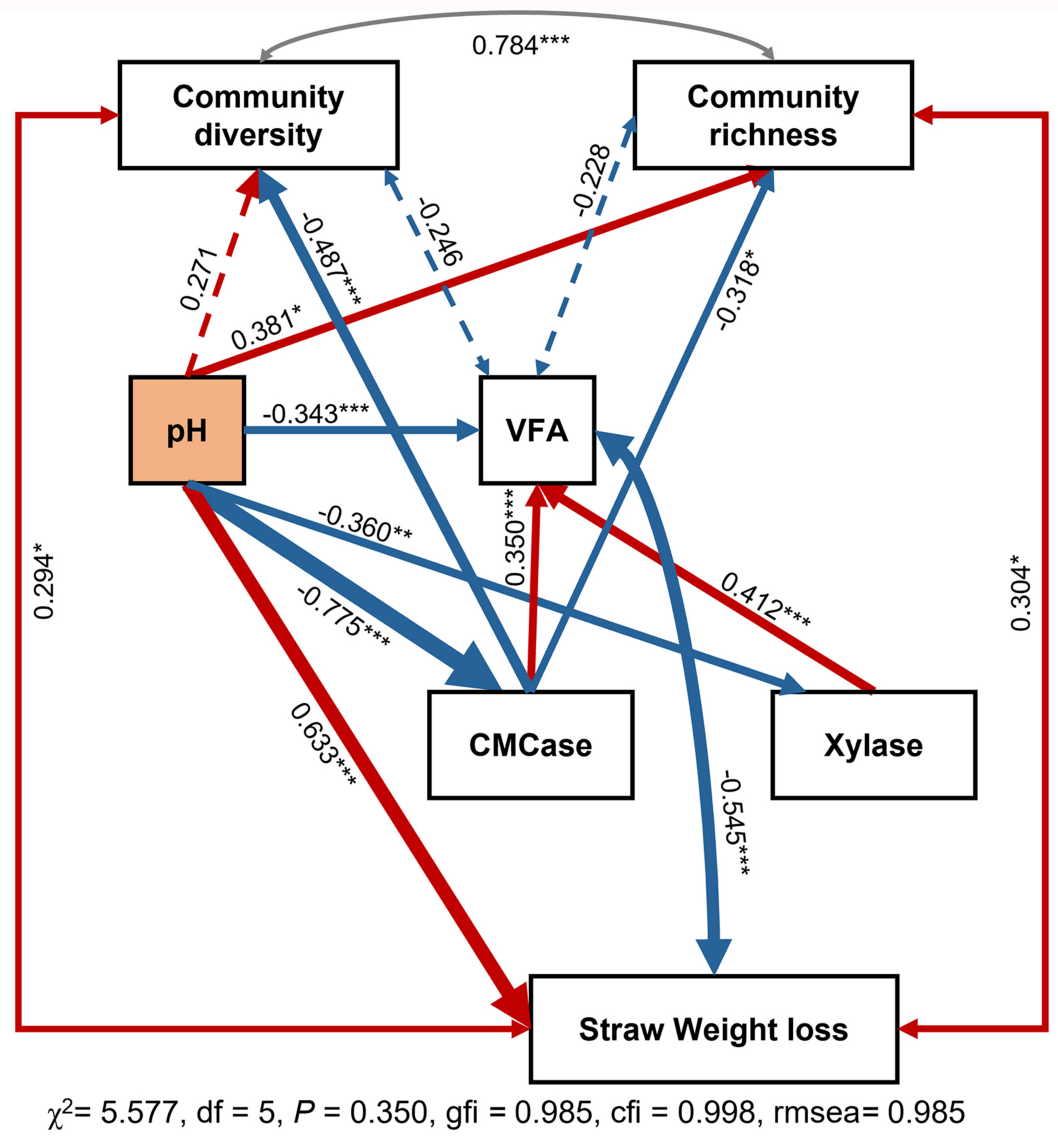
SEM analysis of the relationships among environmental variables, community diversity, and soil function. The width of the arrows is proportional to the strength of the relationship, with red lines indicating a positive correlation and blue arrows indicating a negative relationship. Community diversity is calculated using the Shannon index, while community richness is calculated using the ACE index. *P < 0.05, **P < 0.01 and ***P < 0.001.

To better understand the underlying mechanisms driving the relationship between community composition and soil function, a conceptual scheme and structural equation modeling were constructed (Figure 7). Results showed that neither CMCase nor Xylase had a direct impact on straw degradation in paddy soil. Additionally, straw degradation had weak correlations with soil community diversity and richness. Notably, pH had a significant impact on the microbial community, soil enzyme activity, and strawweight loss, highlighting the driving role of pH in these processes.

## 4 Discussion

### 4.1 Microbiome interaction during lignocellulose degradation in paddy soil

In our study, the robust lignocellulose degradation observed in paddy soils, even without microbial addition or stimulation, points to the presence of highly efficient lignocellulose-degrading microbes inherently present in the soil. This reservoir of microbial diversity serves as a valuable resource for potential applications. When considering the priming effect, implicated in the decomposition of challenging organic matter, our results suggest the involvement of distinct microbial groups during the biodegradation of the lignocellulosic complex, such as rice straw, introduced into the soil during the experiment.

Microbial analysis unveiled a dominant microbiome in paddy soils, characterized by Firmicutes, Actinobacteria, Proteobacteria, and Chloroflexi phyla, aligning with findings from previous studies (Wang et al., 2021, 2022). However, noteworthy differences were observed in the relative proportions of these microbial groups. As reported by Cortes-Tolalpa et al. (2016), the assembly of microbial communities can vary significantly with different inocula, yet exhibit similar lignocellulose degradation capacities, underscoring the stochastic nature of microbial community assembly (Zhou and Ning, 2017).

This reinforces the intricate dynamics of microbial communities in responding to the introduction of complex organic matter, shedding light on the nuanced interactions between microbial populations and challenging substrates like lignocellulose. Such insights are critical for understanding the broader ecological implications of lignocellulose degradation in soil ecosystems.

The results of microbial co-occurrence networks and module analyses revealed that microorganisms in the soil community occupy ecological niches defined by different cellulose structural components and participate in lignocellulose degradation (Bastida et al., 2021). This occupation of ecological niches is evident from significant correlations with specific functional traits. Modular analysis of microbial networks provided insights into the competitive interactions among microorganisms in different niches. For instance, module M5, dominated by the phylum Crenarchaeota, exhibited positive correlations with VFA, cellulase (CMCase), and xylanase activities, indicating its involvement in lignocellulose degradation. Module M6, predominantly composed of Actinobacteria, showed positive correlations with VFA and CMCase, further supporting its role in lignocellulose degradation. Module M9 was primarily composed of Actinobacteria and demonstrated a positive correlation with xylanase activity, suggesting its contribution to xylan degradation. Module M3, comprising Actinobacteria, Gemmatimonadota, and Nitrospirota, exhibited negative correlations with CMCase and total nitrogen, suggesting that this module may play different ecological roles in the ecosystem. M1 and M2 exhibited complex interactions, in which multiple microbial species may occupy ecological niches through specialization and cooperation. Notably, the functional differences between M1 and M2 can be attributed to the varying abundances of Firmicutes and Proteobacteria (Figure 6). These findings identified clusters of functionally related or ecologically connected microorganisms, providing a comprehensive view of how microbial communities interact and partition resources in the ecosystem. Understanding these interactions and strategies is crucial for designing effective microbial management strategies and promoting the desired ecological functions in various environments (Xiong et al., 2021).

### 4.2 Effect of bioaugmentation on lignocellulose degradation

Previous studies confirmed the efficacy of composite microbial systems in accelerating lignocellulose degradation (Hu et al., 2019; Wang Y. et al., 2023). In this study, bioaugmentation using a 30-mL XDC-2 culture resulted in enhanced degradation of rice straw during the initial 14 days. However, long-term observations revealed that the CK group without bacterial addition and the BA group exhibited effective rice straw decomposition. Interestingly, despite the differences observed in the initial 14-day decomposition, no significant differences were observed in the microbial community structure of the soil between the control and bioaugmentation groups, which is consistent with the findings of Ecem Öner et al. (2018) in anaerobic digesters.

Based on our observations, we speculate that the indigenous microorganisms present in the rice soil effectively occupied ecological niches related to lignocellulosic degradation. This can be explained by the “priority effect” in community structure formation (Cheong et al., 2021). The lack of significant changes in microbial community structure highlights the importance of considering the ecological niche occupancy of microbial additives when selecting them for use. The ability of microbial additives to occupy ecological niches influences the survival, growth, and interactions of microbes with other organisms in the environment. Failure to effectively occupy an ecological niche can hinder the formation of a stable microbial community and limit desired long-term functional effects. Therefore, it is crucial to evaluate the ecological niche adaptability of additives for their successful application.

### 4.3 Effect of biostimulation on lignocellulose degradation

In oligotrophic environments, the addition of nutrients can stimulate the growth of indigenous microorganisms and enhance soil decomposition capacity (Bastida et al., 2021; Liu B. et al., 2021). However, according to the “Black Queen” hypothesis, excessive nutrient input can lead to the degeneration of microbial structures (Morris et al., 2012; Oliveira et al., 2014). In the present study, we observed a scenario in paddy soils that closely resembled this. That is, in the high-nutrient group, we observed upregulation of functions related to nutrient metabolism, such as chemoheterotrophy, fermentation, and aerobic chemoheterotrophy (Figure 3C). As the consumption capacity of public goods increases, their functional ability to decompose complex substrates, such as straw, to produce public goods weakens. In the high-nutrient group, the ability to decompose straw and the corresponding enzyme activity significantly declined. This phenomenon suggests that with ample public goods (e.g., easily accessible nutrients), microorganisms may shift their focus from complex substrate degradation to the consumption of readily available resources. Consequently, the efficiency of straw degradation and the production of related enzymes diminishes in nutrient-rich environments. The supply of readily available nitrogen also led to a decline in functions associated with nitrogen transformation, including nitrification, aerobic ammonia oxidation, nitrate reduction, and nitrogen respiration (Figure 3C).

Microbial diversity analysis revealed a decrease in the alpha diversity of the soil microbial community structure following the addition of readily available nutrients (Figure 2B). Moreover, microbial network analysis revealed a decrease in the number of network nodes, accompanied by an increase in network connectivity, suggesting that microbial supplementation enhanced the internal connections within the community while reducing its breadth (Figure 5). Collectively, these findings suggest that nutrient supplementation strengthens internal community interactions while reducing community diversity, resulting in a trade-off between enhanced specific functions and a decline in functional diversity. Similarly, Liao et al. (2021) found that N fertilization enhances the internal interactions among saprotrophic fungi, which benefit from the increased availability of organic substrates while suppressing N-fixing bacteria and fungi, which are less competitive under high N conditions. Regarding the limitations of this study, we did not investigate low-nutrient conditions. However, identifying this critical threshold in future studies would improve our understanding of the impact exerted by exogenous nutrient addition on soil microbes and their functions.

### 4.4 The driver role of pH during lignocellulose degradation

Further analysis of soil physicochemical factors using SEM revealed the decisive role of pH in the rice straw degradation process (Figure 7). In the experimental groups with neutral pH values (CK and BA), the degradation rate of straw exhibited a reasonable positive correlation with the CMCase and xylanase enzyme activities. However, in the acidified groups (BE and BAE), the degradation rate was negatively correlated with CMCase and xylanase enzyme activities (Figure 4). Notably, these crucial physicochemical factors are often overlooked in correlation analyses. Owing to their significant influence, their effects tend to exhibit polarized differentiation, reflecting only extreme values of imbalance and normality without an intermediate normal distribution on a local scale. In our experiment, this manifested as the absence of significant correlations between pH values and degradation rates when conducting differential analysis solely in the low-nutrient group with normal pH or the high-nutrient group with acidified pH. Similarly, network analysis demonstrated this phenomenon, which can lead to spurious results if appropriate grouping factors are not considered. Without considering critical environmental factors like pH, the results obtained from the correlation analysis and microbial correlation network analysis would be mere disturbances rather than true biological correlations. Similar observations were reported in the study conducted by Zhang et al. (2017), who investigated the impact of fertilization on soil bacterial communities. They revealed that soil pH is the most critical factor influencing bacterial community structure in acidic and near-neutral soils but not alkaline soil. This highlights the importance of considering specific soil pH conditions when studying the relationships between soil properties and microbial communities. Only when key environmental factors such as pH are included can the conclusions derived from the correlation and microbial correlation network analyses hold genuine biological significance.

pH is a key edaphic factor that regulates the distribution and function of bacterial communities in various soil environments, such as red soil (Muneer et al., 2022), mountain soil (Tian et al., 2021), and Arctic soils (Feng et al., 2014). Herein, we identified pH as an important indicator for examining the stability of the microbial community structure and function during lignocellulose degradation. These findings suggest a potential strategy for dealing with possible soil acidification during the process of straw return to the field. For example, we can maintain the physiological activity of the soil by increasing its pH to achieve a better straw-return effect. One application of this theory is straw burning or the artificial addition of wood ash (Bang-Andreasen et al., 2017). However, it should be noted that further field testing is needed to verify these speculations and better guide production practices.

## 5 Conclusions

Paddy soil harbors highly efficient lignocellulose-degrading microorganisms, offering valuable resources. This study reveals the significant lignocellulose degradation potential of paddy soil, even without adding exogenous microbes. Microbial analysis elucidated the community structure, highlighting collaborative interactions within the ecological niches formed by cellulose components. Bioaugmentation experiments demonstrated the feasibility of enhancing degradation. However, nutrient enrichment poses the risk of degenerating the community structure, wherein pH has emerged as a crucial factor. Thus, this study emphasizes the importance of pH in microbial community manipulation. Overall, these findings contribute to our understanding of lignocellulose degradation in rice soil and emphasize the potential of native microorganisms. Future research should explore functional diversity and interactions to optimize the degradation efficiency and support sustainable biomass utilization. These insights will provide guidance for harnessing lignocellulose degradation in soil ecosystems.

## Data availability statement

The datasets presented in this study can be found in online repositories. The names of the repository/repositories and accession number(s) can be found below: https://www.ncbi.nlm.nih.gov/sra/PRJNA1010947.

## Author contributions

YW: Conceptualization, Writing – original draft, Writing – review & editing. YC: Conceptualization, Investigation, Writing – original draft. XG: Data curation, Methodology, Writing – original draft. MC: Data curation, Formal analysis, Writing – original draft. QW: Software, Writing – original draft. DZ: Writing – review & editing. PG: Conceptualization, Funding acquisition, Writing – review & editing.
