# Supplementary material for: Unveiling the driving role of pH on community stability and function during lignocellulose degradation in paddy soil
Source: Front Microbiol. 2024 Feb 26;15:1338842. doi: 10.3389/fmicb.2024.1338842 (PMC10925614; doi:10.3389/fmicb.2024.1338842)
Supplement: Supplementary file 1 [file Data_Sheet_1.docx]

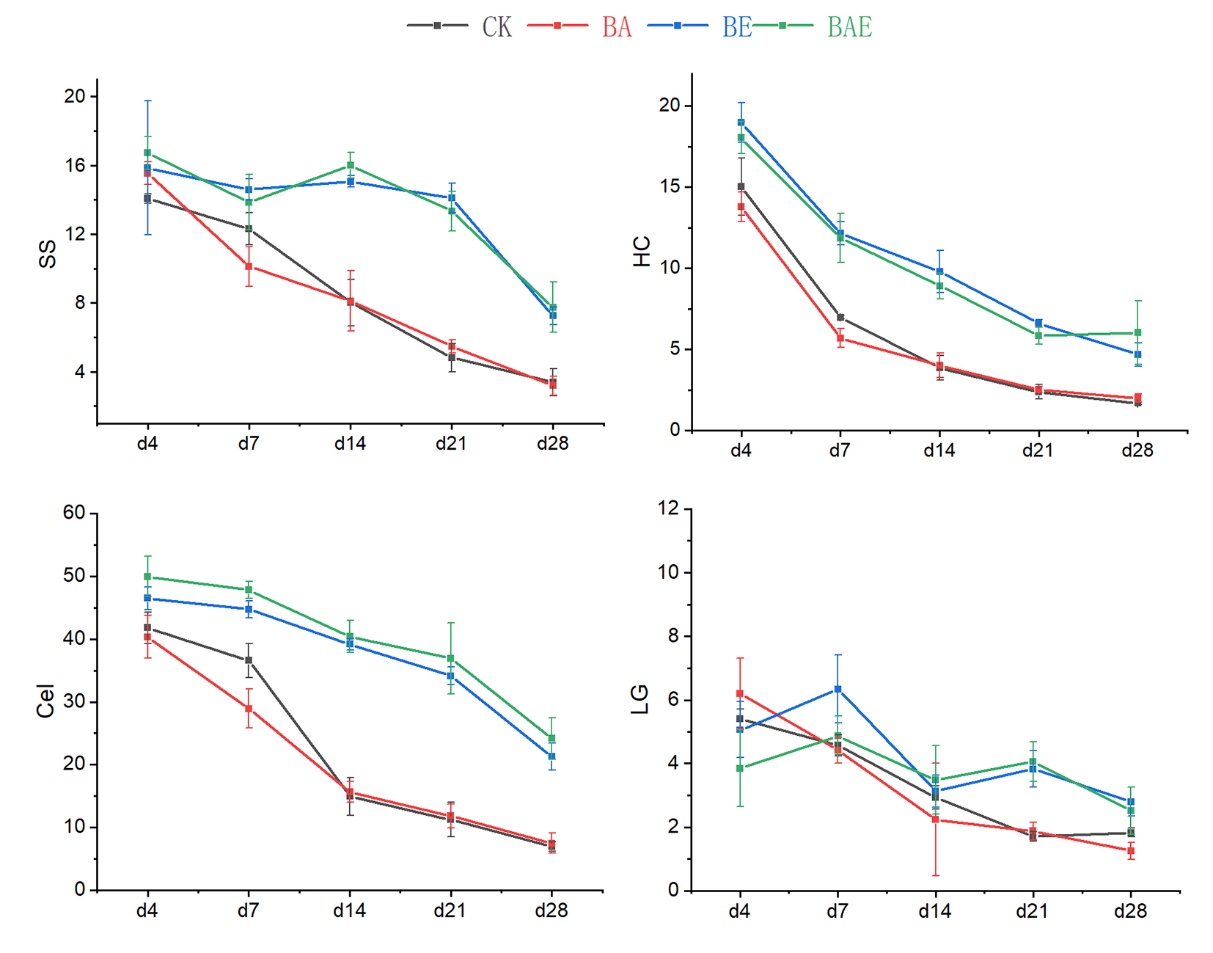


**Figure S1. The changes of lignocellulose components in rice straw.** The data represent the mean and standard deviation from triplicate independent experiments. The changes in the components of lignin (LG), soluble substances (SS), cellulose (Cel), and hemicellulose (HC) were consistent with the weight loss of the rice straw.
